# Encephalitozoon hellem infection after haploidentical allogeneic hematopoietic stem cell transplantation in children: a case report

**DOI:** 10.3389/fimmu.2024.1396260

**Published:** 2024-05-28

**Authors:** Yue Shang, Yuanyuan Ren, Lipeng Liu, Xia Chen, Fang Liu, Xiaolan Li, Yang Wan, Wenbin An, Wenyu Yang, Xiaofan Zhu, Ye Guo

**Affiliations:** ^1^State Key Laboratory of Experimental Hematology, National Clinical Research Center for Blood Diseases, Haihe Laboratory of Cell Ecosystem, Institute of Hematology and Blood Diseases Hospital, Chinese Academy of Medical Sciences and Peking Union Medical College, Tianjin, China; ^2^Tianjin Institutes of Health Science, Tianjin, China

**Keywords:** Encephalitozoon hellem, HSCT, child, mNGS, GVHD

## Abstract

**Background:**

Encephalitozoon hellem (E. hellem) infection is a zoonotic disease, rarely observed in individuals, causing various clinical manifestations including diarrhea, keratoconjunctivitis, cystitis, etc. E. hellem infection after hematopoietic stem-cell transplantation (HSCT) is a rare, serious complication.

**Case presentation:**

Herein, we present a case of E. hellem infection developing during HLA-haploidentical HSCT in a 9-year-old boy who suffered from aplastic anemia. On 15 days after HSCT, the patient developed recurrent and prolonged fever, diarrhea and hematuria. It is challenging to differentiate whether the symptoms mentioned in this case are caused by graft-versus-host disease (GVHD) or a specific infection. Based on the result of metagenomic next-generation sequencing (mNGS) and clinical observation, the patient was diagnosed as E. hellem infection, and received albendazole and decreased the immunosuppressive treatment. Finally, he had recovered.

**Conclusion:**

We should pay attention to the uncommon disease caused by the E. hellem infection after HSCT, especially in cases with immune reconstitution unrecovered. Among those rare infection, mNGS can be performed for better understanding the source of infection and targeted therapy, which can benefit the patients.

## Introduction

The Encephalitozoon hellem infection is a zoonotic disease with a low prevalence, mostly occurring in HIV patients, with a few reports of its occurrence in immunocompromised individuals such as solid organ transplant recipients and cancer patients. According to published reports, Encephalitozoon hellem infections have been documented in various clinical manifestations including diarrhea, keratoconjunctivitis, sinusitis, bronchitis, nephritis, cystitis, etc (1).

Graft-versus-host disease (GVHD) is a prevalent immune complication that can occur after allogeneic hematopoietic cell transplantation (alloHCT), representing a significant health problem in these patients, and being associated with high morbidity and mortality. As widely acknowledged, GVHD is caused by the recognition and the destruction of the recipient tissues and organs by the donor immune effector cells, targeting various organs include the skin, the lower and upper gastrointestinal tract as well as the liver (2).

It is challenging to differentiate between GVHD and Encephalitozoon hellem infection due to their overlapping intestinal symptoms and involvement of multiple organs; notably, the latter is infrequently encountered. Here, we will report the first case of E. hellem infection in a child after haploidentical allogeneic hematopoietic stem cell transplantation (HSCT).

## Case description

A 9-year-old boy, with a history of thrombocytopenia for over 5 years, has been receiving oral cyclosporine treatment for nearly 4 years without significant improvement. As a result, he was admitted to the hospital for further evaluation and management.

Based on the results of pancytopenia and bone marrow smear and biopsy suggesting hypoplasia, he was diagnosed with aplastic anemia. Later second-generation sequencing revealed that the affected child carried a specific genetic mutation called DKC1 (NM_001363.5;intron9;c.915 + 10G>A,chrX:153997595) from the mother, but the pathogenicity or disease-causing potential of this mutation remained uncertain. Furthermore, cytogenetic examination revealed a normal male karyotype. And he didn’t have a PNH clone. Taken together, being ineffective on immunosuppressive therapy, the child was diagnosed with aplastic anemia that required regular blood transfusions. Unfortunately, there was no fully matched sibling donor available. Considering no available sibling-matched donor, he received granulocyte-colony stimulating factor (G-CSF)-mobilized peripheral blood stem cell and bone marrow stem cell from his human leukocyte antigen (HLA) 7/12 matched father on September 7, 2023, after signing a fully informed consent form.

To prepare for the transplantation procedure, the child underwent a conditioning regimen. Patients received 0.8 mg/kg intravenous busulfan (Bu) four times per day on days −8 and −7, 30mg/m2 intravenous fludarabine (Flu) once per day from days -8 to -4, 50 mg/kg intravenous cyclophosphamide (Cy) once per day on days -3, -2 and 2.5 mg/kg rabbit antithymocyte globulin (ATG) once per day on days from days -6 to -2. Additionally, GVHD prophylaxis consisted of tacrolimus, mycophenolate mofetil (MMF), and short-term methotrexate (MTX). Tacrolimus was started as a continuous intravenous infusion on day −5 at a dose of 0.01mg/kg per day and switched to oral administration of 0.5mg daily on day +30, adjusting the drug dosage to maintain a target blood concentration of 4–12 ng/mL. From day -8, MMF (500 mg per 12 hours) was administered orally at a dose based on body weight (30mg/kg/day), and it was tapered to half dose on day +18 and discontinued on day +27. MTX was given on day +1 at a dose of 15 mg/m2 and 10 mg/m2 on days +3, +6, and +11. The patient has been prescribed acyclovir and letermovir for antiviral prophylaxis, trimethoprim-sulfamethoxazole for prophylaxis against Pneumocystis pneumonia, and posaconazole for antifungal prophylaxis following transplantation. On day +10, a peripheral blood analysis using short tandem repeats (STR) markers showed complete donor cell chimerism, and neutrophil and platelet engraftment occurred at 11 days and 12 days, respectively.

**Figure 1:**
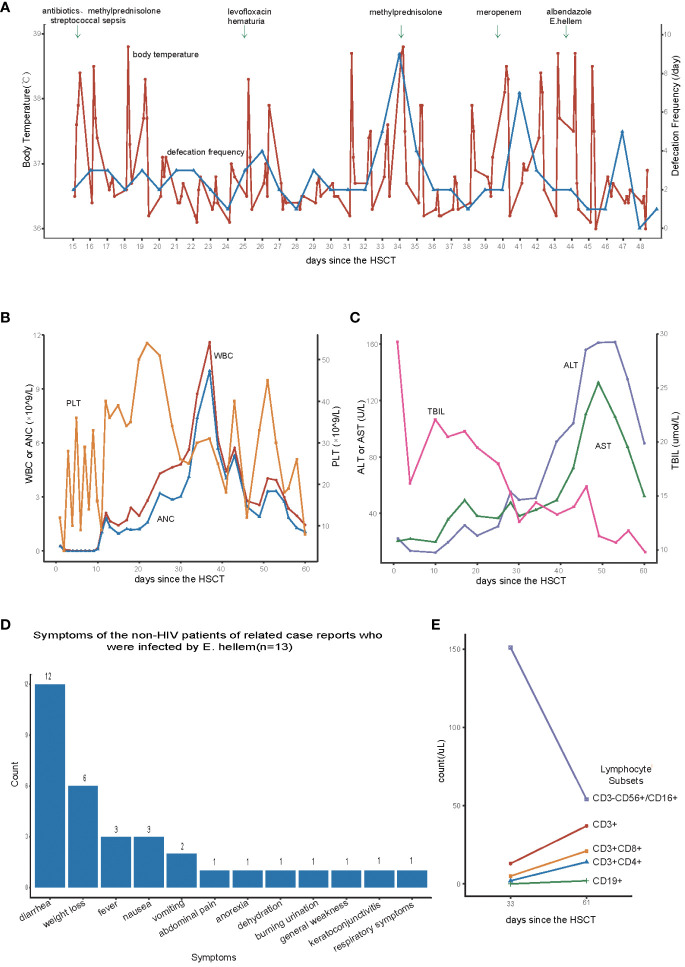
**(A)** Changes in body temperature and defecation frequency after hematopoietic stem cell transplantation. **(B)** Changes in white blood cells(WBC), neutrophils(ANC), and platelets(PLT) after hematopoietic stem cell transplantation. **(C)** Changes in aspartate aminotransferase (AST), alanine aminotransferase (ALT), and total bilirubin levels (TBIL)after hematopoietic stem cell transplantation. **(D)** Symptoms of the non-HIV patients of related case reports who were infected by Encephalitozoon hellem **(E)** Lymphocyte subpopulation count after hematopoietic stem cell transplantation.

The patient had a fever 15 days after the transplant, characterized by a mild peak temperature occurring every 12 to 24 hours. The highest body temperature fluctuated between 37.9 to 38.8°C, without rashes. The illness lasted for nearly a month, with intermittent periods of temperature improvement followed by relapse (**Figure 1A**). Additionally, the patient also had streptococcal sepsis in the early stage (+15day) and was treated with a full course of antibiotics for the positive bacterial infection. Afterward, the patient experienced a temporary decrease in temperature, and the blood bacterial culture yielded negative results. On day +34, the child still had a fever, accompanied by mild nausea, decreased appetite, and insignificant weight loss. To treat GVHD, methylprednisolone was added at a dose of 1mg/kg, resulting in improved gastrointestinal symptoms and body temperature fluctuations within the normal range. However, after reducing the dose of methylprednisolone on day +38, the body temperature rose again, following the same fever pattern as before. Since 15 days after the transplantation, the patient experienced yellow mushy stool, with a frequency of 1–3 times per day (**Figure 1A**), totaling approximately 200ml. Treatment with oral rifaximin and montmorillonite powder did not yield significant improvement. On day +34, the defecation frequency slightly increased to 9 times per day, with each stool being yellow, watery or loose/mushy in consistency, and approximately 700ml in volume. Several stool tests for bacterial and fungal cultures, CMV, EBV, Clostridium difficile, as well as microscopic examination for parasites (roundworm eggs, whipworm eggs, hookworm eggs, etc.), all yielded negative results. Adjusting the treatment for GVHD did not improve the characteristics of the stool. At the same time, the patient developed hematuria, frequent urination, and decreased urine volume starting from 25 days after the transplant. Despite treatment including aggressive hydration, alkalinization, diuresis and levofloxacin for anti-infection did not improve the symptoms, which gradually worsened. The urine BK virus DNA count was 22,700,000 copies. Liver enzyme/bilirubin tests during this period remained within the normal range (**Figure 1C**). On day +44, peripheral blood metagenomic next-generation sequencing (mNGS) revealed 48,321 copies of E.hellem. What’s more, gram stain examination of the stool showed yeast-like fungal spores, but no hyphae were observed (**Supplementary Figure 1**). Therefore, we highly suspect the child was infected with E.hellem, leading us to discontinue the antibiotics and reduce the immunosuppressive drugs. At the same time, albendazole was administered orally at a dose of 0.4g twice a day. Two days later, the body temperature returned to normal, and four days later, the stool returned to normal. Besides, the symptoms of cystitis improved compared to before. The child continued to take oral albendazole at a dose of 0.4g twice a day for 30 days and then reduced to a maintenance dose of 0.2g twice a day. After 2 months, the medication was stopped, and the symptoms of cystitis improved. Based on the result of mNGS and the positive response to adjusted treatment, we believe that the patient is infected with E. hellem. Currently, it’s been seven months since the transplant. The patient is free from fever, diarrhea, or other manifestations of GVHD, and the condition remains stable. Regular follow-up was conducted outside the hospital.

## Discussion

The lack of full immune recovery is a significant factor contributing to the susceptibility of post-transplant patients to infections. It is important to have a correct understanding of infection and GVHD to provide patients with the most effective treatment, which is a crucial clinical issue.

In this case, the patient’s low level of lymphocyte subsets after transplantation (**Figure 1E**) led to the susceptibility of Encephalitozoon hellem infection. The patient initially presented with fever as the main symptom, with a peak temperature fluctuating around 38°C every 12 to 24 hours. Blood culture reported streptococcus mitis, but no clear infection focus was identified. We discontinued the use of the central venous catheter and administered a course of antibiotics to target the positive bacteria. However, there was no significant improvement in fever symptoms, and the fever pattern remained unchanged. Concurrently, the patient experienced mild nausea, loss of appetite, and diarrhea, which are digestive symptoms. At this point, the patient has already undergone hematopoietic reconstruction, and there is a slight upward trend in the patient’s liver enzymes and total bilirubin levels (**Figure 1B**). Consequently, we suspected the presence of acute-GVHD (aGVHD). However, despite initiating frontline corticosteroid treatment, the patient did not exhibit significant improvement, and the condition persisted. Routine blood, urine, and stool cultures, as well as CMV and EBV virus tests, did not provide evidence of infection. The patient may be suffering from a rare pathogenic infection. The mNGS is a high-throughput nucleic acid sequencing-based technique used for pathogen identification in clinical specimens. It does not require cultivation and can rapidly and accurately detect various pathogens. It is an indispensable tool for assisting diagnosis in complex infection patients undergoing allo-HSCT (3). Therefore, we sent the patient’s peripheral blood for mNGS testing to further clarify the source of infection. The mNGS results suggested that the patient was infected with E.hellem, which is a rare pathogenic infection and has not been previously reported in HSCT.

Microsporidia is a kind of eukaryotic organism that is an obligate intracellular parasite. It parasitizes various animals, including humans, and can be transmitted through food and water, causing zoonotic diseases (1). The Encephalitozoon species, including Encephalitozoon intestinalis, Encephalitozoon hellem, and Encephalitozoon cuniculi, are widely distributed in mammals and have been most commonly seen in immunocompromised patients (e.g., patients with HIV infection, especially in patients with CD4 counts of <50/mm3, transplant recipients, and those receiving immunomodulatory therapy) (1). It is not clear what the incubation period of E. hellem is. The earliest reported case of microsporidia infection in a patient after HSCT was not further classified due to limited diagnostic methods (4). However, with the development of diagnostic techniques, microsporidia can be identified using traditional histopathological staining, transmission electron microscopy, staining microscopes, as well as immunological and molecular biological detection methods. Encephalitozoon hellem infection has been reported in patients with AIDS, solid organ transplant recipients, leukemia, lymphoma, hemodialysis, and malignant solid tumors (5–9). We reviewed several case reports of non-HIV patients infected with E. hellem (6, 7, 10), totaling 13 cases. The most common clinical manifestation was diarrhea, followed by weight loss, nausea, and fever (**Figure 1D**). The patient’s main symptoms during the course of the disease were recurrent fever, diarrhea, hematuria, involving the digestive and urinary systems, and in order to screen for other foci of infection, two thoracic CTs were performed, both of which suggested interstitial lung lesions, and there were no significant changes compared with the pre-transplantation period. And urinary ultrasound suggests thickening of the bladder wall, which was consistent with his cystitis presentation. Besides, there is no new pathognomonic change in head MR, and the patient also had no complaints of ocular discomfort. Dinesh (11) reported that microsporidia are one of the main causes of intestinal infection in HIV-positive children. HIV-infected children usually have low immune function, while this patient’s early T-cell subset and B-cell subset decreased significantly after transplantation, indicating slow immune reconstitution and susceptibility to various pathogens. Therefore, in addition to considering the poor efficacy of empirical anti-infective treatment, it is also necessary to consider the possibility of a rare microsporidia infection causing mild fever and gastrointestinal symptoms. Additionally, the patient experienced symptoms of hemorrhagic cystitis (grade 3) during the disease process, including frequent urination, dysuria, and hematuria. Compared with other patients, the clinical manifestations were significantly more severe. Treatment with hydration, alkalinization, diuresis, and hemostasis did not show obvious efficacy. The urinary symptoms had improved after roughly eight days of albendazole use (+52d), but the copies of BKV at that time was still high. The copies of the virus in urine didn’t dropped apparently(< 7 log_10_ copies/mL)until 89 days after HSCT. We speculated that this may also be related to E. hellem infection, as gastrointestinal and urogenital symptoms improved only after treatment for parasitic infection.

In terms of E. hellem infection, Nourrisson (6) reported multiple cases of patients showing significant improvement after treatment with albendazole and a reduction in the original immunosuppressants, leading to a cure in almost all patients. In this case, the infected child also quickly recovered from fever and diarrhea symptoms after receiving albendazole treatment, and the symptoms of cystitis were alleviated as well. Nevez (7) reported a case of E. hellem infection in a patient with CD4+ T-cell prolymphocytic leukemia, which also caused symptoms of cystitis. Weber (12) found that an HIV patient infected with E. hellem experienced mild hematuria. Due to technical and economical limitations, we were unable to definitively identify E. hellem in the child’s urine sample. However, considering the recurrent history of the disease and the significant improvement in symptoms after albendazole treatment, it is plausible to consider that the urinary system symptoms in the child are either caused by or exacerbated by E. hellem infection. Furthermore, we observed that although the child’s platelets were successfully implanted early on, they declined during the later stages of the infection, fluctuating between 12 to 54 × 10^9/L (**Figure 1B**). However, the degree of chimerism between the child and the donor remained complete. We speculate that the poor engraftment of platelets in the child may be related to the persistent infection in the early stages of transplantation.

The symptoms of the patient in this case were quickly relieved after the application of albendazole. The dosage was reduced after one month and discontinued after two months. However, it remains uncertain whether the optimal duration and long-term use of medication may bring other problems, considering the rare occurrence of E. hellem infection following HSCT. Continued follow-up is still necessary. Besides, because of the relatively expensive tests and the patient’s financial reasons, we were unable to send stool or urine mNGS or peripheral blood PCR to confirm that Encephalitozoon hellem infection was indeed a limitation of our report. Based on this case, the consideration of rare E. hellem infections after HSCT is also needed, as fever, diarrhea, and aggravated cystitis may be related to E. hellem infection after transplantation. Furthermore, the mNGS helps to identify the source of infection and facilitate targeted therapy, which can greatly benefit the patients.

## Data availability statement

The original contributions presented in the study are included in the article/**Supplementary Material**. Further inquiries can be directed to the corresponding author.

## Ethics statement

The studies involving humans were approved by the Ethics Committee and Institutional Review Board of Institute of Hematology and Blood Diseases Hospital, Chinese Academy of Medical Sciences and Peking Union Medical College. The studies were conducted in accordance with the local legislation and institutional requirements. Written informed consent for participation in this study was provided by the participants’ legal guardians/next of kin. Written informed consent was obtained from the minor(s)’ legal guardian/next of kin for the publication of any potentially identifiable images or data included in this article.

## Author contributions

YS: Writing – original draft. YR: Writing – review & editing. LL: Writing – review & editing. XC: Writing – review & editing. FL: Writing – review & editing. XL: Writing – review & editing. YW: Writing – review & editing. WA: Writing – review & editing. WY: Writing – review & editing. XZ: Writing – review & editing. YG: Writing – review & editing.
