# Supplementary material for: Encephalitozoon hellem infection after haploidentical allogeneic hematopoietic stem cell transplantation in children: a case report
Source: Front Immunol. 2024 May 28;15:1396260. doi: 10.3389/fimmu.2024.1396260 (PMC11165047; doi:10.3389/fimmu.2024.1396260)
Supplement: Supplementary file 1 [file DataSheet_1.docx]

## Supplementary Figures


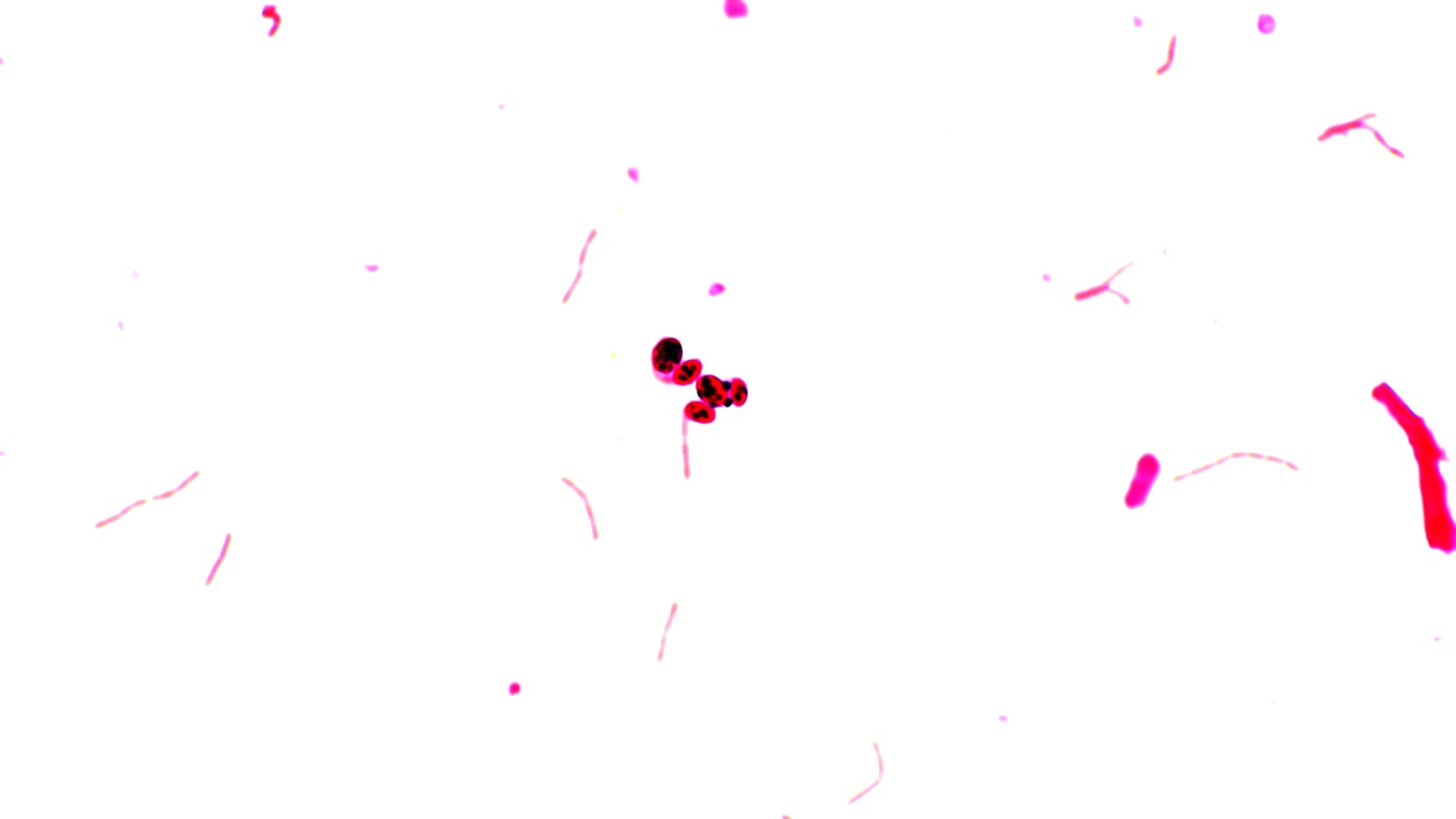


**Supplementary Figure 1.** Direct Gram stain microscopic examination of stools (×1000)
